# Involvement in State and Federal Aging Policy

**DOI:** 10.1093/geroni/igaa057.2385

**Published:** 2020-12-16

**Authors:** Erica Solway

**Affiliations:** University of Michigan, Ann Arbor, Michigan, United States

## Abstract

The fourth speaker is Dr. Erica Solway. Dr. Solway will discuss her experience working in policy at the federal level as a Health and Aging Policy Fellow and a policy advisor with the U.S. Senate Committee on Health, Education, Labor, and Pensions Subcommittee on Primary Health and Aging and in her current involvement in policy-relevant research at the state and national level at the University of Michigan Institute for Healthcare Policy & Innovation. Dr. Solway is a senior project manager forthe evaluation of the Healthy Michigan Plan, Michigan’s Medicaid expansion program, and is also an associate director of the University of Michigan National Poll on Healthy Aging.

